# Targeting the *Cryptococcus neoformans* var. *grubii* Cell Wall Using Lectins: Study of the Carbohydrate-Binding Domain

**DOI:** 10.3390/molecules20033776

**Published:** 2015-02-25

**Authors:** Pamella de Brito Ximenes, Eduardo Isidoro Carneiro Beltrão, Danielle Patrícia Cerqueira Macêdo, Maria Daniela Silva Buonafina, Reginaldo Gonçalves de Lima-Neto, Rejane Pereira Neves

**Affiliations:** 1 Department of Mycology, Universidade Federal de Pernambuco (UFPE), Av. Prof. Nelson Chaves, s/n°-Cidade Universitária, Recife 50670-420, Brazil; E-Mails: pamella_ximenes@hotmail.com (P.B.X.); danielabuonafina@hotmail.com (M.D.S.B.); 2 Department of Biochemistry, Universidade Federal de Pernambuco (UFPE), Av. Prof. Nelson Chaves, s/n°-Cidade Universitária, Recife 50670-420, Brazil; E-Mail: ebeltrao@hotmail.com; 3 Department of Pharmaceutical Sciences, Universidade Federal de Pernambuco (UFPE), Av. Prof. Nelson Chaves, s/n°-Cidade Universitária, Recife 50670-420, Brazil; E-Mail: daniellemacedo28@gmail.com; 4 Department of Tropical Medicine, Universidade Federal de Pernambuco (UFPE), Av. Prof. Nelson Chaves, s/n°-Cidade Universitária, Recife 50670-420, Brazil; E-Mail: goncalves_reginaldo@hotmail.com

**Keywords:** lectins, *Cryptococcus neoformans* var. *grubii*, cell wall carbohydrates, diagnosis

## Abstract

*Cryptococcus neoformans* var. *grubii* is considered to be the major cause of cryptococcosis in immunosuppressed patients. Understanding cell wall glycoproteins using lectins is of medical interest and can contribute to specific therapy. The aim of this study was to evaluate the carbohydrates on the cell wall of *Cryptococcus neoformans* var. *grubii* clinical isolates, using a fluorescein isothiocyanate-lectin binding protocol. Thirty yeast strains stocked in the culture collection were cultivated for 2 days at 30 °C with shaking. Cells were obtained by centrifugation, washed in phosphate-buffered saline, and a suspension of 10^7^ cells/mL was obtained. To determine the binding profile of lectins, concanavalin A (Con A), wheat germ agglutinin (WGA), *Ulex europaeus* agglutinin I (UEA-I), and peanut agglutinin (PNA) conjugated to fluorescein were used. All the tested clinical isolates of *Cryptococcus neoformans* var. *grubii* were intensely stained by WGA, moderately stained by Con A, and weakly stained by PNA and UEA-I. Thus, *Cryptococcus* can be detected in clinical specimens such as blood and cerebrospinal fluid using the fluorescent lectin WGA, which may be considered as an option for detection in cases of suspected cryptococcosis with low laboratory sensitivity. Future applications may be developed using this basic tool.

## 1. Introduction

In developed countries, *Cryptococcus neoformans* var. *grubii* is considered to be the major cause of cryptococcosis, a serious human infection that results in death in some cases [1,2,3]. The mechanisms that are involved in its pathogenesis can best be studied by starting with an understanding of cell wall glycoproteins, which are important characteristics of fungi of medical interest [4]. In this context, different lectins have been used to determine a pattern of cellular glycoconjugates in *Cryptococcus* species, including *C. neoformans* var. *grubii* [5,6].

Lectins are a class of proteins that exhibits huge biological and pharmacological potential; lectins were originally identified in plant, animal, and microbe species [7]. These proteins have at least one non-catalytic domain that binds specifically and reversibly to mono- or oligosaccharides [8]. This carbohydrate recognition capacity allows interactions with cell membrane surface glycoconjugates, consequently interfering with various biological events such as infection, cell differentiation, pathogen-host interaction, metastasis, and cell recognition [9,10,11].

A number of studies have consistently demonstrated that a knowledge of the surface components of *C. neoformans* is essential to understand cryptococcosis pathogenesis [5]. It is important to consider that the interaction between the yeast cell wall and host tissue receptors allows the first step in the establishment of disease, adhesion [8]. Thus, the targeting of cell wall glycoconjugates such as *N*-acetyl-d-glucosamine, l-fucose, d-galactose, and glucose/mannose may elucidate many biological and pathological roles that are currently unclear. The expression of *N*-acetyl-d-glucosamine may be analysed using wheat germ agglutinin (WGA), that of methyl-α-d-mannoside using concanavalin A (Con A), the L-fucose using *Ulex europaeus* agglutinin I (UEA-I), and for detection of d-galactose, and glucose/mannose on the cell wall surface, peanut agglutinin (PNA) is considered the most specific lectin [5].

Conventional detection methods for *Cryptococcus* species include culture isolation, colony morphology, and nutritional characteristics. This fungus may take 24–72 h or even weeks to grow in primary culture. For this reason, the diagnosis of cryptococcosis at the fungal species level is time consuming and delays treatment seelction. Furthermore, these methods are laborious and may eventually provide inconclusive identifications [9].

In this context, the aim of this study was to evaluate the expression of *N*-acetyl-d-glucosamine, methyl-α-d-mannoside, l-fucose, d-galactose, and glucose/mannose on the cell wall surface of *C. neoformans* var. *grubii* through staining protocols using wheat germ agglutinin (WGA), concanavalin A (Con A), *Ulex europaeus* agglutinin I (UEA-I), and peanut agglutinin (PNA) lectins.

## 2. Results and Discussion

The lectin-binding assays using WGA, Con A, UEA-I, and PNA showed three carbohydrate-specific fluorescent patterns for the *C. neoformans* var. *grubii* strains as presented in Table 1.

**Table 1:** *Cryptococcus neoformans* var. *grubii* strains and fluorescence patterns of lectin staining.

*Cryptococcus neoformans* var. *Grubii* *URM	WGA lectin	Con A lectin	PNA lectin	UEA-I lectin
5809	+++	++	+	+
5810	+++	++	+	+
5811	+++	++	+	+
5813	+++	++	+	+
5814	+++	++	+	+
5815	+++	++	+	+
5816	+++	++	+	+
5818	+++	++	+	+
5819	+++	++	+	+
5820	+++	++	+	+
5821	+++	++	+	+
5822	+++	++	+	+
5823	+++	++	+	+
5824	+++	++	+	+
5825	+++	++	+	+
6895	+++	++	+	+
6896	+++	++	+	+
6897	+++	++	+	+
6898	+++	++	+	+
6899	+++	++	+	+
6900	+++	++	+	+
6901	+++	++	+	+
6902	+++	++	+	+
6903	+++	++	+	+
6904	+++	++	+	+
6905	+++	++	+	+
6906	+++	++	+	+
6907	+++	++	+	+
6908	+++	++	+	+
6909	+++	++	+	+

Notes: Strains were obtained from the Universidade Recife Micologia Culture Collection of Pernambuco (*URM), Brazil; WGA: wheat germ agglutinin; Con A: concanavalin A; PNA: peanut agglutinin; UEA-I: *Ulex europaeus* agglutinin I.; +++: intense staining pattern; ++: moderate staining pattern; +: weak staining pattern.

**Figure 1:**
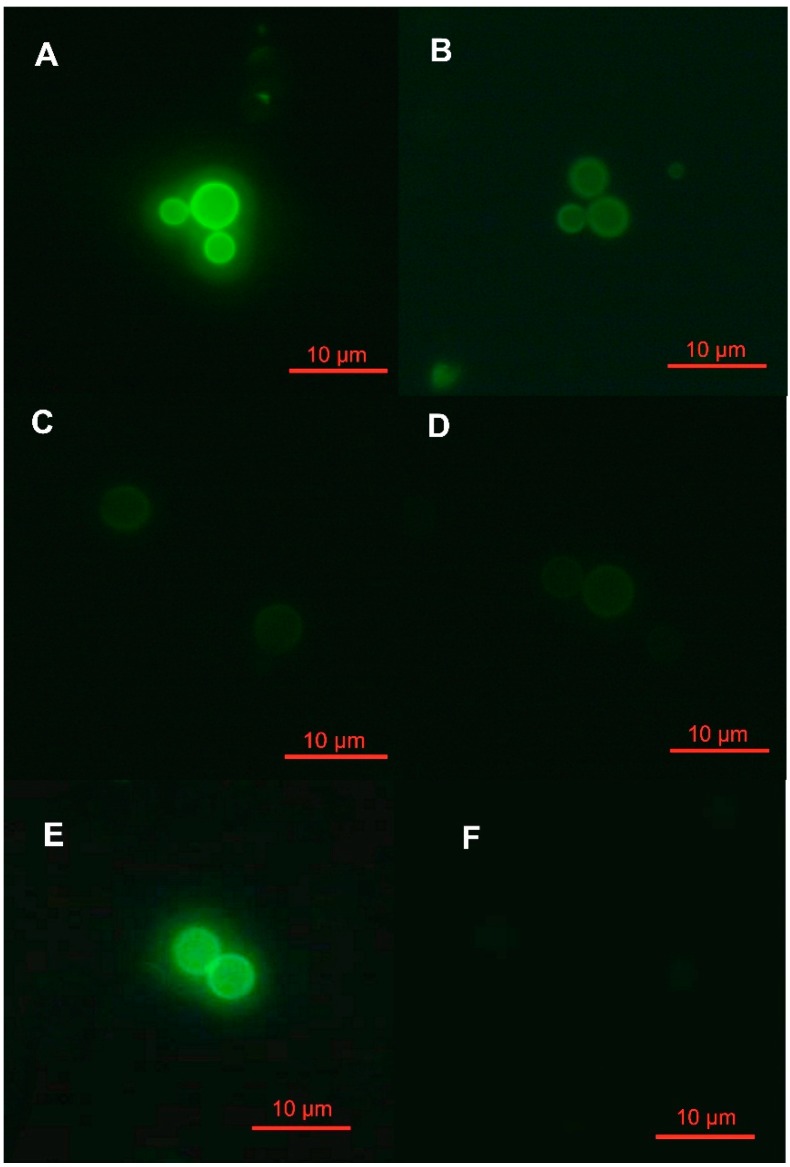
Fluorescence patterns of lectin staining from clinical isolates of *Cryptococcus neoformans* var. *grubii*. The staining was intense (**A**) with wheat germ agglutinin (WGA), moderate (**B**) with concanavalin A (Con A), and weak (**C**,**D**) with peanut agglutinin (PNA) and *Ulex europaeus* agglutinin I (UEA-I). Positive (**E**) and negative (**F**) controls of the fluorescence patterns.

The assays were carried out in duplicate, and the duplicates exhibited the same staining patterns. The patterns were intense, moderate, and weak, respectively (Figure 1).

All of the tested strains of *C. neoformans* var. *grubii* had intense staining with WGA, moderate staining with Con A, and weak staining with PNA and UEA-I when compared with positive and negative controls. These data suggested that *N*-acetyl-d-glucosamine was highly expressed on the cell wall surface of the tested *Cryptococcus* strains because this carbohydrate is the target molecule of WGA. In contrast, moderate and weak expression of methyl-α-d-mannoside and d-galactose/l-fucose were visualized by Con A and PNA/UEA-I staining, respectively.

According to Fonseca *et al.* [10], the intimate association of cryptococcal cell walls with a distinctive enveloping capsule hinders the isolation and purification of cell wall constituents, thereby compromising our understanding of the cryptococcal cell wall composition, structure, and associated biochemistry. Therefore, analysis of the cell wall composition using fluorescent lectin-staining patterns may facilitate the knowledge and perception of these molecules *in situ*.

Previous studies indicated that a typical basidiomycete fungal cell wall (as verified in *Cryptococcus* species) predominantly comprises *N-*acetyl glucosamine in the form of chitin microfibrils with gel-like glycoproteins, many of a mannose nature [11]. The *Cryptococcus* capsule is predominantly comprised of mannose and has been shown to elicit and modulate immune responses in humans [12,13]. The most common capsular mannose in *Cryptococcus* is the polysaccharide glucoronoxylomannan, which forms up to 88% of the capsular material [13].

Imaging of fluorescein isothiocyanate (FITC)-WGA (Figure 1A), FITC-Con A (Figure 1B), FITC-PNA (Figure 1C), and FITC-UEA-I (Figure 1D) on the cell surface by fluorescence microscopy demonstrated three different staining patterns: intense, moderate, and weak. Based on the results presented in Table 1, the marked fluorescence from the 30 *C. neoformans* var. *grubii* strains exposed to FITC-WGA indicates a high concentration of *N*-acetyl-D-glucosamine at the cell surface. In contrast, moderate fluorescence was detected with FITC-Con A in all of the tested strains. According to Foster *et al.* [13], Con A primarily binds to mannose residues. Additionally, a weak fluorescent pattern was observed in all tested strains when exposed to FITC-PNA and FITC-UEA-I.

Fonseca *et al.* [10] affirm that the abundance of a particular carbohydrate in the *C. neoformans* cell wall is related to the strain and growth phase. Some authors suggest that the nature of the FITC-WGA and FITC-Con A exposure appears to be a defining *Cryptococcus* characteristic [13]. FITC-PNA and FITC-UEA-I exhibited the same results with respect to the fluorescence intensity. Future studies of the *C. neoformans* var. *grubii* carbohydrate composition of the cell wall may select between FITC-PNA and FITC-UEA-I because the staining patterns were similar.

Thus, the detection of this species in clinical specimens such as blood and cerebrospinal fluid using the fluorescent lectin WGA may be considered an option for laboratory analyses, especially in cases of suspected meningitis or in immunosuppressed patients with a deficient serologic response. This basic technique may also be used with flow cytometry to obtain a rapid and reliable diagnosis with characterization of the involved species.

## 3. Experimental Section

### 3.1. Cryptococcus Strains and Growth Conditions

The 30 *C. neoformans* var. *grubii* strains used in this study were obtained from patients diagnosed with cryptococcosis and have been preserved in the Universidade Recife Micologia Culture Collection (Federal University of Pernambuco, Recife, PE, Brazil) since 2008 as a lyophilized material under mineral oil. Initially, the yeast cells were inoculated into 100-mL Erlenmeyer flasks containing 50 mL minimal medium composed of 15 mM dextrose, 10 mM MgSO_4_, 29.4 mM KH_2_PO_4_, 13 mM glycine, and 3 µM thiamine-HCl, pH 5.5. Fungal cells were cultivated for 2 days at 30 °C with shaking. The cryptococcal cells were collected by centrifugation, washed in phosphate-buffered saline (PBS), and counted in a Neubauer chamber to obtain a standard suspension of 10^7^ cells/mL following the method of Rodrigues *et al.* [9].

### 3.2. Cryptococcal Cell Wall Lectin Binding

The binding profile of lectins to the cryptococcal cell wall was verified through the use of Con A, WGA, UEA-I, and PNA conjugated to FITC. First, 1 mL from the previously obtained standard suspension of the yeast cells in PBS was centrifuged at 470 g in microfuge tubes for 3 min. Following this step, the pellet was suspended in 0.1% trypsin for 3 min at 37 °C and washed again by centrifugation in PBS. This procedure was repeated twice. After this step, the 25 µg/mL ConA, WGA, UEA, and PNA lectins conjugated to FITC (FITC-lectin) were added separately to the yeast cells obtained after sequential washes for incubation at 4 °C for 1 h. The preparation was centrifuged, and the pellet was washed with PBS to evaluate the lectin specificity patterns using a fluorescence microscope (AXIO Imager M2m, Zeiss, Calgary, AB, Canada). The binding profiles were expressed according to the fluorescence intensity as follows: intense, +++; moderate, ++; and weak, +. All procedures were repeated twice.

Particularly for the use of WGA lectin, one additional step was developed to prepare samples for binding evaluation. This step involved an enzymatic pre-treatment with neuraminidase to prevent non-specific binding to sialic acids. Specifically, 100 µL neuraminidase (Sigma, St. Louis, MO, USA) was added to the yeast suspension after the washes and incubated for 1 h at 37 °C before the addition of the lectin according to the previously described steps.
